# Metformin Promotes Osteogenic Differentiation of Adipose-Derived Stromal Cells and Exerts Pro-Osteogenic Effect Stimulating Bone Regeneration

**DOI:** 10.3390/jcm7120482

**Published:** 2018-11-26

**Authors:** Agnieszka Śmieszek, Krzysztof A. Tomaszewski, Katarzyna Kornicka, Krzysztof Marycz

**Affiliations:** 1 Department of Experimental Biology, The Faculty of Biology and Animal Science, University of Environmental and Life Sciences, 50-375 Wroclaw, Poland; kornicka.katarzyna@gmail.com (K.K.); krzysztof.marycz@upwr.edu.pl (K.M.); 2 Department of Anatomy, Jagiellonian University Medical College, 12 Kopernika Street, 31-034 Krakow, Poland; krzysztof.tomaszewski@uj.edu.pl; 3 Faculty of Veterinary Medicine, Equine Clinic-Equine Surgery, Justus-Liebig-University, 35392 Gießen, Germany

**Keywords:** adipose-derived multipotent stromal cells, metformin, osteogenic differentiation, bone healing

## Abstract

Metformin, the gold standard in type 2 diabetes treatment, is a drug with multi-faceted effects. Currently, metformin has gained much attention as an agent that may find application in regenerative medicine. In this study, we considered its pro-osteogenic function in the course of in vitro osteogenesis of multipotent stromal cells derived from rat adipose tissue (rASCs). In addition, we evaluated the effect of metformin treatment on bone metabolism in a model of cranial defect in nondiabetic rats. In vitro study showed that metformin that is introduced to the culture medium at concentration equal 500 µM may promote the differentiation of rASCs into bone-forming cells, which express mRNA and secrets proteins that are related to the functional tissue (namely, alkaline phosphatase and osteocalcin). Osteogenic effect of metformin, as determined using in vitro model, was also manifested with the formation of mineralized extracellular matrix rich calcium and phosphorous deposits. We have also found, that in undifferentiated rASCs, metformin significantly activates a critical regulatory factor for osteogenic differentiation, i.e., AMPK. Moreover, using in vivo model we showed metformin administration at a dose of 250 mg/kg/day accelerated bone healing and the formation of mature tissue at a fracture site in rat cranial defect model. The obtained results shed promising light on metformin application in regenerative orthopedics, both as an agent improving functionality of ASCs for therapeutic transplantation, as well as a medication enhancing the bone healing process.

## 1. Introduction

Bone fractures are a significant health problem—for the individual and for the society alike. High fracture risk is associated with various civilization disorders e.g. diabetes, obesity, and osteoporosis [1]. The scale of the problem is also magnified by age-related diseases, because of an increasing life expectancy of the population [2,3]. Bone regeneration is associated with mobilization of various cell populations to the fracture site. Multipotent stromal cells (MSCs) contribute to this process by supporting regeneration through differentiation or paracrine activity [4]. The bone-building capacity of multipotent stromal cells and their application for regenerative orthopedics, in the majority was investigated in regards to MSC derived from bone marrow (BMSCs) or the mononuclear cell fraction of bone marrow [5]. However, an attractive alternative for BMSCs is MSCs that are isolated from adipose tissue (ASCs). The main reasons for this are ASCs availability (easy surgical access), excellent expansion, and proliferation capacities, but foremost the low level of senescence of ASCs in long term in vitro culture [6]. However, BMSCs, when compared with ASCs, exhibit superior osteogenic potential. Thus, there is a great need for biological and/or chemical agents that would accelerate ASC osteogenic differentiation in vitro [5,7]. Obtaining sufficient amounts of osteogenic progenitors from adipose tissue could help to restore bone structure and function, lost due to trauma or disease.

Regardless of the development in MSCs based therapies, novel directions of regenerative medicine are aimed at seeking pharmaceutical agents that will improve the osteogenic capacity of MSC transplants, as well as bone regeneration potential [8]. Metformin, a drug that is commonly used for diabetes mellitus treatment has been proposed as a good chemical agent in terms of pro-osteogenic function, both in vitro and in vivo [9,10,11,12]. The pleiotropic character of metformin is now a well-established fact—for example it was shown that metformin may reduce cardiovascular, nephropathy, and cancer risk in diabetic patients [13,14,15,16]. The use of metformin is also associated with a decreased risk of fracture in diabetic patients [17].

The pro-osteogenic properties of metformin have been investigated in vitro using both pre-osteoblasts cell line models (mouse MC3T3-E1 and rat UMR-106), as well as rat bone marrow progenitor cells [9,11,18,19]. Metformin enhances cell proliferation and promotes their osteogenic differentiation reflected in the increase of type I collagen (Coll-1) production, alkaline phosphatase (ALP) enzymatic activity, and bone morphogenetic protein-2 (BMP-2) expression. One of the main conclusions of the in vitro studies that analyze metformin-related osteogenic activity is that the promotion of cells proliferative activity and their osteogenic differentiation seem to be strictly dependent on metformin concentration [9,11,18,19]. Metformin also influences extracellular matrix mineralization, which is associated with AMP-activated protein kinase (AMPK), an enzyme that plays a key role in lipid metabolism [11]. In addition, metformin may govern the cross-talk between AMPK and serine-threonine protein kinase Akt pathways [20]. Activity of Akt and AMPK are inversely correlated, as was demonstrated by the Pantovic et al. who showed that AMPK may control osteogenic differentiation of human MSC’s through late activation of Akt [21]. It seems that the protective effects exerted on bone tissue might be associated with the anti-adipogenic effects of metformin [22]. Additionally, our previous research showed that administration of metformin in mice may negatively influence ASCs’ proliferation and alter adipose tissue structure—the source of ASCs. Moreover, metformin, in a dose-dependent manner, inhibited the expression and production of osteopontin by mice ASCs [23]. In this context, the anti-proliferative effect of metformin may also be essential for modulation of ASCs osteogenic differentiation in vitro.

The in vivo effect of metformin on bone metabolism is still under debate—study results vary depending on the animal model and metformin dose used. The number of reports utilizing the rat model indicates that metformin treatment may positively influence bone density and quality [10,11]. Our recent findings, using a mouse model, also support the thesis regarding the pro-osteogenic properties of metformin [24]. However, contradictory opinions can also be found in the works of Jeyabalan et al. [25] and Hegazy et al. [26]. Discrepancy in this scientific area indicate a strong need for further research in terms of potential advantages of metformin on bone formation and healing.

Since metformin was reported as an agent inducing osteogenic differentiation of BMSCs in a dose-dependent manner, in the present study we decided to determine its effect on ASCs. We hypothesized that metformin, at a defined concentration, may also trigger in vitro osteogenic differentiation of ASCs. The overarching goal of our experiment was to address the role of metformin in fracture healing. We investigated the pro-osteogenic properties of metformin supplementation in rat re-ossification model of minimal bone lesion in healthy animals. The obtained results shed promising light on metformin application in regenerative orthopedics, both as an agent improving functionality of ASCs for therapeutic transplantation, as well as a medication enhancing the bone healing process.

## 2. Experimental Section

This study was conducted with the approval of the Second Local Bioethics Committee at the Department of Biology and Animal Breeding, Wroclaw University of Environmental and Life Sciences, Wroclaw, Chelmonskiego 38C, Poland (December number 177/2010 from 15 November 2010). All reagents used in this experiment were purchased from Sigma-Aldrich (Poznan, Poland), unless indicated otherwise.

### 2.1. Isolation and Culture of Rat Adipose-Derived Multipotent Mesenchymal Stromal Cells (rASC)

Six, four-month-old male Wistar rats were obtained from the Animal House of Wroclaw Medical University (Wroclaw, Poland). Animals were sacrificed by an intraperitoneal injection of morbital (Biowet, Pulawy, Poland). Subcutaneous adipose tissue (2 g) was collected from each animal and immediately placed in sterile Hank’s balanced salt solution (HBSS). Adipose-derived multipotent mesenchymal stromal cells were isolated under aseptic conditions, by cutting the adipose tissue into small pieces and digesting it with collagenase type I (1 mg/mL) for 40 m in an incubator at 37 °C and 5% CO2. The tissue was centrifuged at 1200× *g* for 10 m, the supernatant was removed, and the pellets with cells were re-suspended in Dulbecco’s Modified Eagle’s Medium (DMEM) containing Ham’s F-12 nutrient mixture supplemented with 10% of fetal bovine serum (FBS) and 1% of antibiotic/antimycotic solution, and then placed in a T-25 culture flask. The cells were cultured at constant conditions in a CO**_2_** incubator (37 °C, 5% CO2, and 95% humidity) throughout the experiment. Subsequent cultures were maintained in a complete growth medium (CGM) i.e., DMEM with high concentration of glucose −4500 mg/L supplemented with 10% FBS and 1% antibiotic solution. Passage was performed when culture reached 70–80% of confluence. To obtain a number of cells sufficient for performing the experiment, the cultures were passaged three times. 

### 2.2. Characterization of rASCs

#### 2.2.1. Immunophentotype

For immunophenotyping, cells at second passage were cultured on a 24-well plate, with a seeding density of 2 × 10^4^ cells per well. Cells’ phenotype was assessed when the cultures reached 60% confluence. The procedure was performed accordingly our well-established protocol [27]. Briefly, analysis cells were fixed using 4% PFA for 45 m in room temperature, washed three times with HBSS, and permeabilized for 15 m with HBSS containing 0.2% Tween 20 and 5% of goat serum. Following permeabilization, the cultures were washed again using HBSS. Primary anti-body labeling was performed in HBSS overnight at 4 °C. The following primary antibodies were employed: HCAM CD44 antibody, 5′-nucleotidase—CD73 antibody (1:200 dilution), glycosylphosphatidylinositol-anchored glycoprotein—CD90 antibody (1:20 dilution), endoglin—CD105 antibody (1:100 dilution), and CD45—protein tyrosine phosphatase, C type receptor antibody (1:100 dilution; Abcam, Cambridge, UK).

All anti-rat primary antibodies were produced in rabbit. After specific staining, cells were washed with HBSS and incubated for 1.5 h in room temperature with secondary antibody in HBSS—goat anti-rabbit IgG conjugated with Atto448 (1:800 dilution). Antibody dilutions were used in accordance with the manufactures’ protocols. Observations of stained cultures were performed using an inverted microscope (Axio Observer A.1, Zeiss, Oberkochen, Germany) and documented using a Power Shot digital camera (Canon, Woodhatch, UK).

#### 2.2.2. Multipotency Assay

To confirm the multipotent character of rASC, the cultures at third passage underwent osteogenic, chondrogenic, and adipogenic differentiation. The specific stimulation of rASCs was performed using commercially available kits (StemPro**^®^**, Life Technologies Polska Sp. z o.o., Warsaw, Poland). Cultures that were maintained at CGM served as a reference. The assay cells were seeded into 24-well plates at inoculum 2 × 10^4^ cells per well. Differentiation of rASCs towards osteoblasts lasted 21 days, while into adipocytes and chondrocytes 16 days. Functional matrix formation was evaluated, i.e., (i) osteogenic cultures were stained with Alizarin Red in order to reveal calcium deposits and to determine osteogenesis process efficiency; (ii) nodules rich in cartilage specific proteoglycans were detected with Safranin O; and, (iii) lipid droplets in adipogenic cultures were evaluated using Oil Red O. All staining procedures were performed according to the manufacturer’s protocols. Preparations were analyzed using inverted microscope (Axio Observer A.1, Zeiss, Oberkochen, Germany) and documented using a Power Shot digital camera (Canon, Woodhatch, UK).

#### 2.2.3. The Proliferative Activity 

Proliferative potential of rASCs was determined using the resazurin-based assay. The test was performed according to the manufacturer’s protocol. For the analysis, cells at third passage were inoculated into 24-well plates, with an initial concentration of 2 × 10^4^ cells per well, suspended in 0.5 mL of complete growth medium. The analysis was performed at 24 h, 48 h, 96 h, 120 h, and 168 h of cells’ culture. To evaluate the population doubling time an online software was used [28] Proliferation activity assays were performed in triplet for each cell population isolated. To assess the clonogenic potential of rASCs, the CFU-F assay was performed. Cells at third passage were placed in six-well dishes at initial inoculum equal to 100 cells per well. After seven days of culture, cells were fixed using 4% PFA and were stained with pararosaniline. Routinely, three CFU-F assays were performed for each rASCs population. Colonies that were formed by more than 30 cells were counted under an inverted microscope (Axio Observer A.1, Zeiss, Oberkochen, Germany) and documented using a Power Shot digital camera (Canon, Woodhatch, UK).

#### 2.2.4. Morphology and Growth Pattern 

Cultures analyzed under an epifluorescence microscope were fixed in 4% PFA for 45 m at room temperature. Next, each culture was washed three times with HBSS and permeabilized with 0.2% Tween 20 in HBSS for 15 m. Phalloidin—atto488 (1:800 dilution) staining was used to visualize actin filaments, whereas diamidino-2-phenylindole (DAPI, 1:1000 dilution) to observe cell nuclei. Staining results were documented under an inverted microscope (Axio Observer A.1, Zeiss, Oberkochen, Germany) and were documented using a Power Shot digital camera (Canon, Woodhatch, UK). Culture evaluation in scanning electron microscope (EVO LS15, Zeiss, Oberkochen, Germany) was also performed after culture fixation with 4% PFA for 45 m at room temperature, however after the cultures were fixed, the ethanol series dehydration was performed. Dehydrated samples were air dried and sputter-coated with gold for 250 s (Scancoat Six, HHV Ltd., Crawley, UK). Observations were performed using an SE1 detector at 10 kV of filament’s tension.

### 2.3. Effect of Metformin on rASCs Osteogenic Cultures-In Vitro Model

#### 2.3.1. Experimental Cultures with Metformin 

The rat adipose-derived multipotent mesenchymal stromal cells (rASCs) used for the experiment were isolated from six, four-month-old male Wistar rats and were characterized in terms of their cellular phenotype, its ability to differentiate into osteogenic, adipogenic, and chondrogenic precursors. The morphology and proliferative activity of rASCs was also determined. For the in vitro test, rASCs at the third passage were inoculated at a concentration of 2 × 10^4^ into a 24-well culture plate. Osteogenic cultures were propagated using a medium dedicated for multipotent stromal cells differentiation into osteoblast (StemPro^®^, Life Technologies Polska Sp. z o.o., Warsaw, Poland). Cells cultivated in non-osteogenic conditions served as control for osteogenesis efficiency evaluation. Both control and experimental cultures were carried out in triplicate. Metformin hydrochloridum (Metformax^®^ 850; Teva Pharmaceuticals Polska Sp. z o.o., Warsaw, Poland) was dissolved in the growth media at 10 μM, 50 μM, 100 μM, 500 μM, and 1000 μM concentrations. Cell incubation with metformin begun after 24 h when about 90% of inoculated cells were adhered. The culture media were refreshed every two days. The proliferation activity of cultures was determined after the 1st, 7th, 14th, and 21st day of culture using the resazurin-based test. The analysis was performed according to the method described previously [29]. To monitor changes in cell proliferation in osteogenic cultures the proliferation factor (PF) was assessed. The normative value of PF reflected the activity of rASCs in osteogenic cultures in relation to the control cultures, therefore a PF > 1 expressed an increase in cellular activity, whereas a PF < 1 a reduction of the proliferation rate.

#### 2.3.2. Efficiency of Osteogenesis in rASCcultures with Metformin

Expression of osteogenic markers was analyzed both on the mRNA, as well as protein level. Total RNA was isolated using the acid guanidinium thiocyanate-phenol-chloroform extraction method [30]. Cultures were rinsed with HBSS and homogenized in TriReagent^®^. Total RNA (150 ng) was treated with DNase I RNase-free kit (Thermo Fisher Scientific, Waltham, MA, USA) and reversed transcribed with Tetro cDNA Synthesis Kit (Bioline Reagents Limited, London, UK). Enzymatic digestion of total RNA and cDNA synthesis were performed in accordance with the manufacturers’ instructions using a T100 Thermal Cycler (Bio-Rad, Hercules, CA, USA). Real-time PCR was carried out using the SensiFAST SYBR^®^&Fluorescein Kit SYBR green PCR kit and the CFX Connect Real-Time PCR Detection System (Bio-Rad, Hercules, CA, USA). Primer sequences and PCR conditions are summarized in Table S1 of Supplementary Materials. The quantity of target genes in osteogenic cultures where normalized to the reference gene, related to a control culture, and expressed as fold change (2^−ΔΔCt^), as described by Livak and Schmittgen [31]. 

The activity of alkaline phosphatase was accessed in the supernatants collected after 7 and 14 days of culture. The supernatants from each culture were prepared in triplicates and were diluted two-fold. The substrate was hydrolyzed into p-nitrophenol by alkaline phosphatase. The measurement was performed using an Alkaline Phosphatase Colorimetric Assay Kit (Abcam, Cambridge, UK) and the p-nitrophenyl phosphate (pNPP) was used as a phosphatase substrate of the reaction. The procedure was described in detail previously [32]. The reaction product was measured at 405 nm wavelength using a spectrometer (BMG Labtech, Ortenberg, Germany). Sample readings were then applied to the standard curve to obtain the amount of pNP that was generated by the ALP sample. The enzymatic activity was determined using the following formula: ALP activity (µU/mL) = A/V/T, where: (i) A is the amount of pNP generated by the samples (in µmol); (ii) V is the volume of sample added to the assay well (in mL), and (iii) T is the reaction time.

The analyzed osteogenic markers i.e., Gla-osteocalcin (OCL) and bone morphogenetic protein 2 (BMP-2), as well as leptin (LEP) were detected in the supernatants that were collected after 21 days of each cell culture. The presence of OCL was determined using Rat Gla-Osteocalcin High Sensitive EIA Kit (Takara, Otsu, Japan), the secretion of BMP-2 was evaluated using BMP-2 Quantikine ELISA Kit (R&D Systems, Minneapolis, MN, USA), while leptin secretiton was measured using Mouse/Rat Leptin Quantikine ELISA Kit (R&D Systems, Minneapolis, MN, USA). Each sample was tested in triplicate for the presence of specific markers. The quantitative determination of investigated proteins was performed according to the manufacturers’ instructions. Sample absorbance was measured at 450 nm. The amount of detected proteins was expressed as ng/mL of supernatant.

Mineralized nodules were visualized with Alizarin Red. The staining procedure was performed according to the manufacturer’s instruction (Sigma Aldrich, Poznan, Poland). Stained cultures were observed under an inverted microscope (Zeiss, Axio Observer A.1) and documented using the Canon Power Shot Camera. The pictures were analyzed using ImageJ and Pixel Counter plugin (version 1.6.0, U. S. National Institutes of Health, Bethesda, MD, USA), as it was described previously [33] using minimum insensitivity value established at 200 relative units. SEM imaging (Zeiss Evo LS 15) was performed on post-fixed cells, rinsed with distilled water, and dehydrated by graded series of ethanol (from 50% to 100%, using 10% interval). The elemental composition of the extracellular matrix was measured using scanning electron microscopy with energy dispersive X-ray spectroscopy (SEM-EDX). The Quantax detector (Evo LS 15, Zeiss, Oberkochen, Germany; Quantax, Bruker, Coventry, UK) was used for the evaluation of calcium and phosphorous deposits—obtained values were presented as weight percentages (wt%). The number of osteogenic nodules that were formed in the experimental cultures were determined using ImageJ software (NIH; http://rsb.info.nih.gov/), while the morphometry of nodules was assessed using a SEM platform. 

#### 2.3.3. Mechanisms of Metformin Action

In order to determine the influence of metformin on AMPK and AKT activation and phosphorylation, two specific ELISA-based test were used—AMPKa Total and Phospho T172 In-Cell ELISA Kits (Abcam, Cambridge, UK), and Human/Mouse/Rat Phospho-Akt (S473) Immunoassay (R&D Systems, Minneapolis, MN, USA). Assays were performed following manufactures’ recommendations. For the test, rASCs at the third passage were inoculated into 96-well plates at a concentration equal 30,000 cells per well. Before the experiment cells were serum-starved for 2 h and incubated with either 500 µM of metformin or 500 µM of AICAR (positive control) for 60 m. Obtained results were presented as normalized values relating to the concentration of phosphorylated molecules to the total protein (AMPK or AKT, respectively). 

### 2.4. The Effect of Metformin on Bone Mass and Fracture Healing in Rats (In Vivo Model)

The experiment was performed on healthy four-month-old male Wistar rats (290 g ± 30 g of body weight). The animals were purchased from the Animal Laboratory House, Wroclaw Medical School, and housed in the Animal Experimental Laboratory (Wroclaw, Poland). Before the experiment animals were allowed to acclimatize for one week after transport. All animals were housed in cages in groups of two and were subjected to a 12 h light/dark cycle with room temperature maintained at 22 ± 2 °C. The experimental model included a group of animals without cranial defect (NCD; *n* = 18) and with cranial defect (CD; *n* = 18). The animals were assigned to the following subgroups: (i) control group (*n* = 6); (ii) metformin-treated group receiving 100 mg/kg of the drug per day (*n* = 6); and, (iii) metformin-treated group receiving 250 mg/kg of the drug per day (*n* = 6). Circular craniotomy defects (1.0 mm in diameter) were drilled according to the method described before by Santana et al. [34]. The defects were created using a cylindrical low-speed carbide bur 1 mm in diameter. Metformin was given in the drinking water, and did not affect the drinking volume. Animals that were assigned to the control group did not receive metformin. The bottles with water were replenished every two days. The experiment was carried out for four weeks. Both water and food were available ad libitum during the experiment period. Body weight of animals was monitored during the experiment and measurements were carried out using electronic weight (RADWAG PS/C1 series, Radom, Poland). At the end of the experiment, the rats were sacrificed by cervical dislocation. Skulls were processed for histology and for micro-CT analysis. Additionally, blood samples were collected for serum biochemical analysis. Blood was collected from animals using the cardiac puncture method [35]. Determination of circulating levels of ALP, OCL, BMP-2, and LEP was performed, as described above. For each analysis, blood serum was diluted five times. Samples were prepared in triplicate for each ELISA measurement. For histological analysis cranial bones were fixed (4% formaldehyde in PBS, 48 h), decalcified in 10% ethylenediaminetetraacetic acid (EDTA) at 37 °C for 5 days. After decalcification samples were rinsed with PBS, and then dehydrated in increasing ethanol gradient of 50%, 60%, 70%, 80%, 96%, and 100%, each incubation was performed for 5 m. Then, the samples were embedded in paraffin. The samples were sectioned transversely (HM 340E Microm, Thermo Fisher Scientific, Waltham, MA, USA) at a thickness of 10 µm, dried, rehydrated in alcohol series, and stained with hematoxylin-eosin and Masson’s trichrome. Next, the samples were dehydrated and mounted under a coverslip. Tartrate resistant acid phosphatase (TRAP) positive cells were detected in deparaffinised bone tissue sections using a commercial acid phosphatase leucocyte kit. The procedure of TRAP staining was performed according to the manufactures’ guidance. Slides that were obtained during histochemical staining were examined using light microscopy (Axio Observer A.1, Zeiss, Oberkochen, Germany). The microcomputed tomography (µCT) measurements of rat skulls were performed using the Nanotom 180N (GE Technologies, Warsaw, Poland). The machine is equipped with a nanofocus X-ray tube with maximum 180 KV voltage. The tomograms were registered on a Hamamatsu 2300 × 2300 pixel detector. The reconstruction of measured objects was performed with the aid of proprietary GE software datosX ver. 2.1.0 using the Feldkamp algorithm for cone beam X-ray CT [36]. The post reconstruction data treatment was performed using VGStudio Max 2.1 [37], Fiji software [38] with the BoneJ plugin [39], and Voxler®3-Golden Software (vesion 4.0) [40]. All examined specimens were scanned at 60 kV of source voltage and 250 μA, with a rotation of the specimen of 360 degrees in 2400 steps. The exposure time was 500 ms and a frame averaging of 6 and image skip of 1 was applied, resulting in a scanning time of 140 m. The reconstructed images had a voxel size of 15 µm^3^. The three-dimensional (3D) reconstruction of each sample was analyzed to determine the amount and shape of newly grown bone. A cylindrical region with the diameter equal to the bone defect drilled in the skull was selected. Any bone inside this region was treated as new bone. The bone fragments adjacent to the selected region but overcrossing it were carefully checked manually to determine their nature. The newly formed bone was extracted as an isolated 3D object. Using 3D solid bool operations, the old skull bone was also extracted. All visualizations were prepared based on those two isolated objects. The volume of newly created bone was calculated using BoneJ [39]. The selected part was also skeletonized (a method of presenting how different bone trabecules link to each other) to determine its topological structure.

## 3. Results

### 3.1. Characteristics of rASC Used in the Experiment

**Figure 1:**
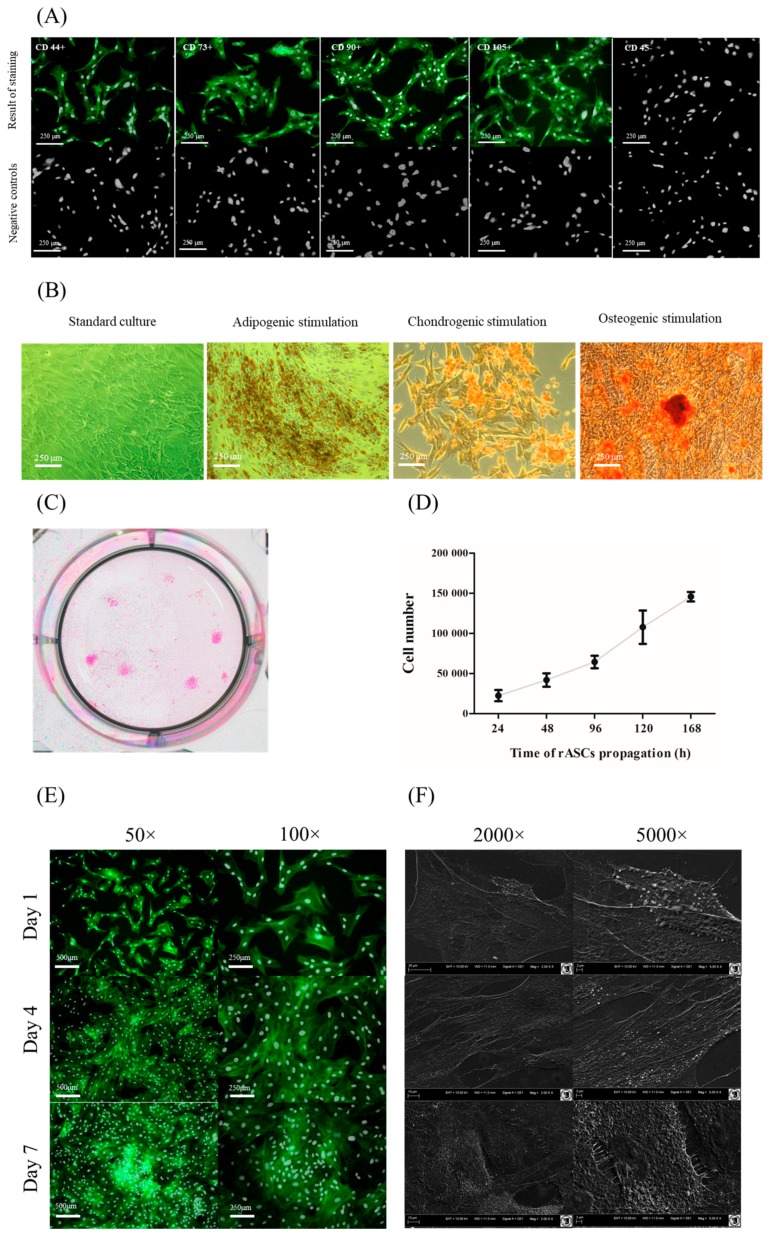
The characteristic of rat adipose tissue (rASC) used in the in vitro experiment. Panel (**A**) the results of immunophenotyping. Analysis was performed on three different ASCs cultures, after the second passage. The obtained cell populations were positive for markers characteristic for mesenchymal cells—CD44, CD73, CD90, and CD105, and were negative for the CD45 hematopoietic marker. Markers were stained with specific primary antibody and secondary antibody conjugated with atto-488 (positive reactions shown in green). Nuclei were stained with DAPI (white dots). Results of negative staining are included in the panel (figures below). Magnification 100×. Scale bar = 250 μm. Panel (**B**) the morphology of rASCs in standard, adipogenic, chondrogenic and osteogenic cultures. The lipid-rich cellular organelles formed after adipogenic stimulation are visible as orange droplets. Chondrogenic nodules are stained with Safranin-O. Calcium deposits formed in the extracellular matrix of the osteogenic culture were stained using Alizarin Red. Images included in the graph were chosen as representative. Both control and experimental cultures were carried out in triplicate. Magnification 100×. Scale bar = 250 μm. Panel (**C**) culture stained with pararosaniline. Panel (**D**) rASCs growth curve related to panel (**C**). Panel (**E**) morphology and growth architecture of rASCs cultures. Nuclei stained with DAPI (white dots), the actin skeleton is stained with phalloidin (green stained cellular bodies). Magnification 100×. Scale bar = 250 μm. Panel (**F**) the formation of cytoplasmic projections and microvesicles shedding. Images depicted in panel (**E**,**F**) are representative and were obtained from cultures carried out in duplicate.

Immunophenotyping of rASCs showed that cells used for the experiment were characterized by the presence of surface markers specific for multipotent mesenchymal stromal cells i.e., CD43, CD73, CD90, and CD105. The cells did not express the hematopoietic origin marker—CD45 (Figure 1A). Additionally, rASCs demonstrated multilineage differentiation potential, and differentiated toward adipogenic, chondrogenic, and osteogenic precursors. Specific staining showed that the propagation of rASCs in the proper differentiation medium promotes the formation of lipid droplets (Oil-Red O), cartilage nodules (Safranin-O), and formation of calcium-rich deposits (Alizarin Red) (Figure 1B). Colony forming unit frequency in rASCs cultures was equal 31% (±7%), while cell population tended to double in 59 ± 1 h. The population of rASCs used in the experiment exhibited exponential growth (Figure 1C,D). In low and semi-confluent cultures (1st and 4th day of rASCs propagation) fibroblast-like morphology of cells was evident. These cultures were morphologically heterogeneous and formed by cells of bipolar or multipolar shapes with centrally located oval nuclei. At the 7th day the fusiform morphology was predominantly due to high culture confluence (Figure 1E). In the early stages of propagation, cells were loosely attached to each other. The formation of dense cellular aggregates was characteristic from the 4th day of culture. Cells at the 4th and 7th day of culture formed well developed intracellular connections. The most abundant microvesicles shedding was noted at the1st and 4th day of rASCs culture (Figure 1F).

### 3.2. Osteogenic Cultures with Metformin

#### 3.2.1. Metabolic Activity

**Figure 2:**
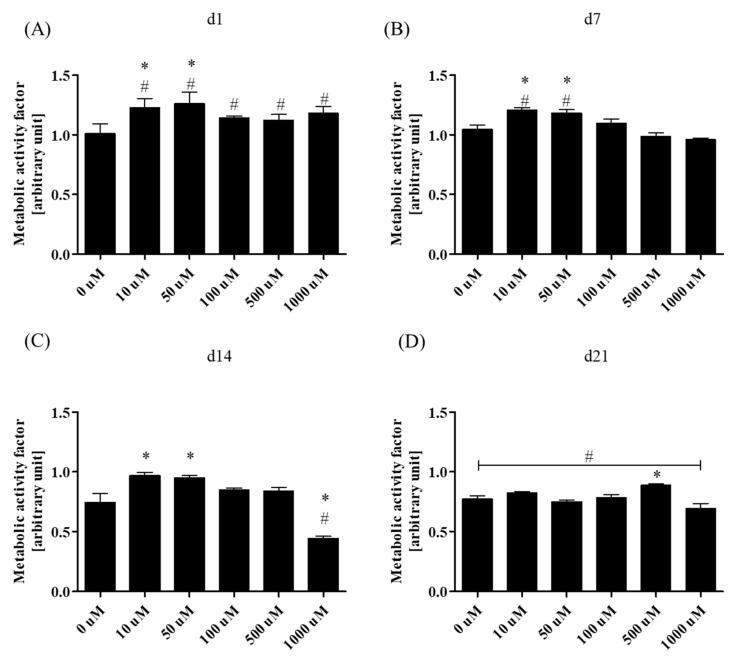
Metabolic activity of rASCs in osteogenic (OG) cultures treated with metformin. The metabolic activity was monitored after 24 h of culture (d1, **A**), as well as after 7- (7d, **B**), 14- (14d, **C**) and 21- days (d21, **D**) of culture. Metabolic activity factor was determined in respect to the metabolic activity of the non-osteogenic cultures. Statistical analysis, related to multiple comparisons, was performed with regard to the control culture—metformin non-treated, and was indicate with an asterisk (*). The differences in metabolic activity between rASCs in osteogenic and non-osteogenic cultures were indicated with hashtag (#). The statistical analysis were performed at *p* < 0.05.

Metabolic activity of rASCs was monitored for 21 days in both osteogenic (OG) and non-osteogenic cultures (Non-OG). The metabolic activity factor of osteogenic cultures was determined during the essential phases of osteogenic differentiation in vitro i.e., at the early stage (1st and 7th day of culture), mid-stage (14th day of culture), and late stage (21st day of culture). The rate was determined in relation to the activity of ASCs in non-osteogenic cultures (Figure 2). Obtained results indicate that metformin at concentrations of 10 µM and 50 µM increased the activity of rASCs in osteogenic cultures during the early stage of differentiation. The acceleration of cells metabolic activity in this culture was also enhanced in comparison to the non-osteogenic conditions. During the mid-stage and late-stage of osteogenic cultures the metabolic activity of differentiating rASCs was lowered when compared to the non-osteogenic cultures, the difference was statistically significant in the 21st day of culture. However the comparison of metabolism in OG cultures revealed that in the 14th day the 10 µM and 50 µM of metformin increased cells activity when compared to the cultures without metformin, while the highest concentration decreased it. The metabolic activity of rASCs in late-osteogenesis supported by 500 µM of metformin was also increased.

#### 3.2.2. Bone-Associated Markers’ Gene Expression Analysis

**Figure 3:**
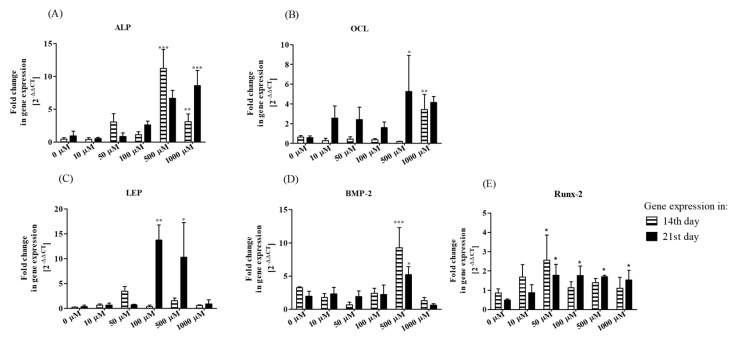
The influence of metformin on the expression of mRNA of alkaline phosphatase (ALP, **A**), osteocalcin (OCL, **B**), leptin (LEP, **C**), bone morphogenetic protein-2 (BMP-2, **D**), and Runx-2 (**E**) in rASCs osteogenic cultures. Results of RT-qPCR are presented as relative values, obtained through normalization of transcript levels with the endogenous reference control (GAPDH) and in relation to the expression of target genes in non-OG conditions. Calculations of gene transcripts levels were performed using the 2^−ΔΔCt^ algorithm. Data presented as the mean fold change of relative expression and were compared to control cultures (without metformin); error bars represent standard deviation from the mean calculated for normalized values obtained in three separate reactions; asterisks represent statistically significant differences; * *p* < 0.05; ** *p* < 0.01, and *** *p* < 0.001.

Analysis of ALP gene expression showed that the transcript levels in rASCs osteogenic cultures were affected by metformin at 500 µM and 1000 µM, both in 14th as well as 21st day of culture (Figure 3A). Significant alteration of mRNA level for OCL in the 14-day culture was noted after treatment with 1000 µM of metformin, while in the 21st day of OG cultures the highest expression of the OCL gene was noted when 500 µM of metformin was introduced (Figure 3B). Expression of LEP mRNA was affected only in 21st day of rASCs osteogenic cultures in cases when 100 µM and 500 µM of metformin was used as a supplement (Figure 3C). The transcripts of BMP-2 significantly increased due to treatment with 500 µM metformin, this was noticeable both at the mid-stage and late-stage of in vitro osteogenesis (Figure 3D). The measurement of mRNA level for Runt-related transcription factor 2 (Runx-2) revealed that after 14 days of culture in the osteogenic condition, the transcript level was slightly affected by metformin treatment. The significant increase in mRNA level was noted only in cultures treated with 50 µM of metformin. However, after 21 days of osteogenesis, we noted significantly elevated levels of Runx-2 transcripts in almost all cultures treated with metformin, with the exception in terms of cultures where 10 µM of metformin was introduced. 

#### 3.2.3. Secretory Activity

**Figure 4:**
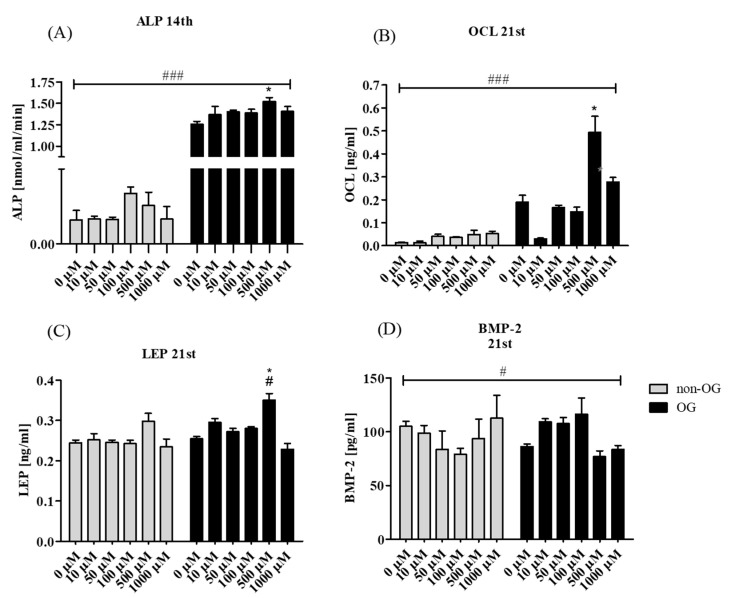
Secretory activity of rASCs, both in non-OG and OG cultures. The alkaline phosphatase (ALP) activity was measured in culture supernatants after 14 days of propagation (**A**). The concentration of osteocalcin (OCL, **B**), leptin (LEP, **C**) and bone morphogenetic protein 2 (BMP-2, **D**) was measured in supernatants collected after 21 day of cultures Statistically significant differences are indicated with an asterisk and hashtag. Statistical analysis, related to multiple comparisons, was performed with regard to the control culture—metformin non-treated, and was indicated with an asterisk (*). The differences in secretory activity of rASCs in osteogenic and non-osteogenic cultures were indicated with hashtag (#). Values are expressed as mean ± SD. Statistically significant differences were noted at *p* < 0.05 (*/#) and *p* < 0.001 (###).

Enzymatic activity of ALP was measured in rASCs cultures at the 7th (Supplementary Materials) and 14th day of differentiation (Figure 4A). Significant influence of metformin on increased ALP activity in OG cultures was noticeable only at the 14th day, the highest ALP activity was noted in OG conditions with 500 µM of metformin. The late marker of osteogenesis OCL was determined in the supernatant that was collected in the 21st day of culture. Increased secretion of Gla-osteocalcin was noted in the osteogenic environment, after metformin treatment at concentration of 500 µM. Interestingly, the amount of OCL decreased in osteogenic cultures when 10 µM of metformin was used (Figure 4B). Leptin level in non-osteogenic cultures was not influenced significantly by metformin treatment. In turn, addition of the drug to the osteogenic medium, within a range of 10 to 500 µM increased extracellular production of leptin during the late stage of rASCs osteogenic differentiation. The most significant increase of leptin was noted in osteogenic cultures that were treated with 500 µM of metformin (Figure 4C). Metformin did not affect the secretion of BMP-2 in non-osteogenic cultures, but in osteogenic environment metformin at concentrations of 10, 50 and 100 µM elevated secretion of BMP-2 in culture supernatants, while the highest concentrations of the drug decreased the BMP-2 concentration. Surprisingly, the BMP-2 secretion was also lowered in OG cultures without metformin (Figure 4D).

#### 3.2.4. Morphological Alternations of rASCs Cultures in the Course of Osteogenic Differentiation

**Figure 5:**
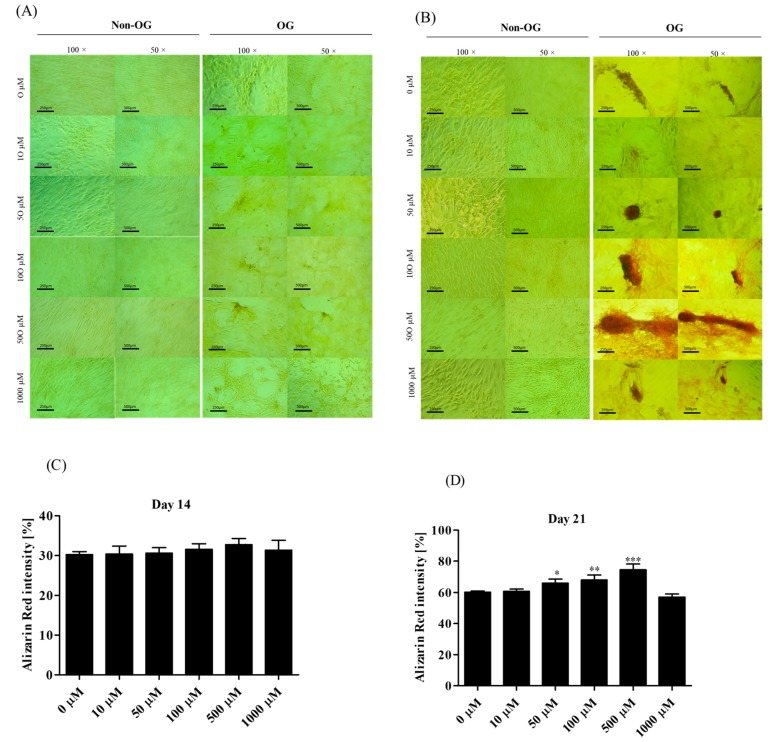
Micrographs presenting the results of Alizarin Red staining of rASCs cultures in non-osteogenic (Non-OG) and osteogenic (OG) conditions. Mineralization of extracellular matrix was determined in 14th (**A**) and 21st day (**B**) of rASCs propagation. Analysis was performed under light microscope. The images were analyzed using ImageJ software in order to determine the Alizarin red staining intensity in OG cultures after 14 day (**C**) and 21 day (**D**). Comparative analysis was performed in relation to the control culture (non-treated with metformin). Asterisks represent statistically significant differences; * *p* < 0.05; ** *p* < 0.01, and *** *p* < 0.001. Presented images were selected as representative across investigated groups. The scale bar for images magnified × 100 = 250 µm, while for images magnified × 50 = 500 µm.

**Figure 6:**
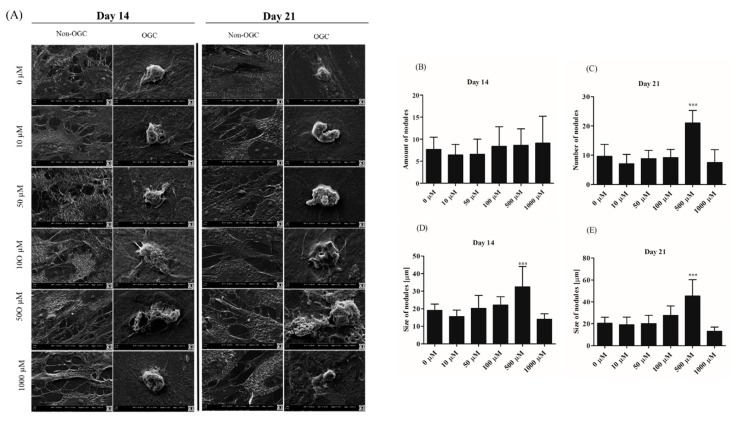
Scanning electron microscopy (SEM) images of rASCs’ non-osteogenic and osteogenic cultures. Magnification 5000×, scale bar = 2 µm (**A**). Nodules formed in osteogenic cultures were counted using ImageJ software (**B**,**C**). The results of morphometric analysis of osteonodules are presented in the panel (**D**,**E**). Statistically significant differences are indicated with an asterisk; *** *p* < 0.001.

**Figure 7:**
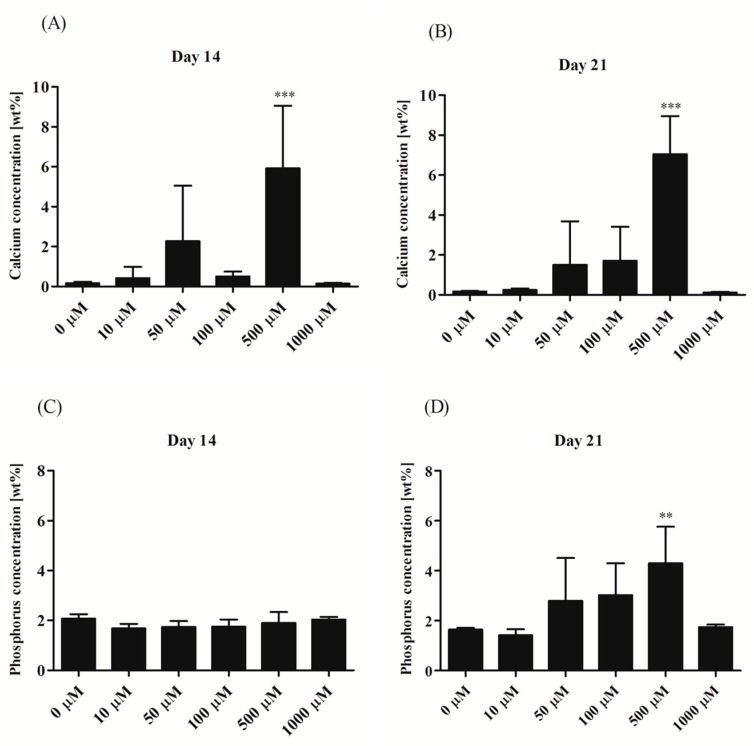
Elemental composition of extracellular matrix formed in osteogenic conditions in rASCs cultures. The elements were measured in extracellular matrix of cultures propagated 14 days (**A**,**C**) and 21 days (**B**,**D**). Calcium and phosphorous content was shown as weight percentage (wt%). Indicated differences were significant at *p* < 0.01(**) and *p* < 0.001 (***).

Analysis of cells’ morphology and culture growth pattern was performed at the 14th and 21st day of rASCs propagation in order to monitor the formation of matrix mineralization (Figure 5A,B). Osteogenic differentiation caused distinguishable changes in cultures, which were associated with the formation of condensed cellular aggregates—osteonodules. The presence of mineralized nodules was visualized with the Alizarin Red S staining and confirmed by the SEM-EDX analysis (Figure 6). Initially, at the 14th day of rASCs differentiation, the number of nodules was comparable between investigated osteogenic cultures. At the final stage of osteogenesis in vitro, the prominent number of nodules was noted in cultures after 500 µM metformin treatment. Additionally, osteonodules that were formed in this culture had the largest diameter, which was already significant at the 14th day of propagation. The analysis of chemical composition of the extracellular matrix in osteogenic cultures showed that the highest content of calcium was noted in cultures that were treated with 500 µM of metformin, both at the 14th and 21st day of propagation. Concentration of phosphorus was comparable in osteonodules that were formed in all experimental cultures after 14 days of propagation. The phosphorous deposition in nodules was influenced by metformin concentrations from 50 µM to 500 µM. In parallel with the increase of calcium deposition, the significant elevation of phosphorus content was noted after 500 µM metformin treatment (Figure 7).

#### 3.2.5. The Effect of Metformin Action on AMPK and AKT Signaling Pathways in rASCs

**Figure 8:**
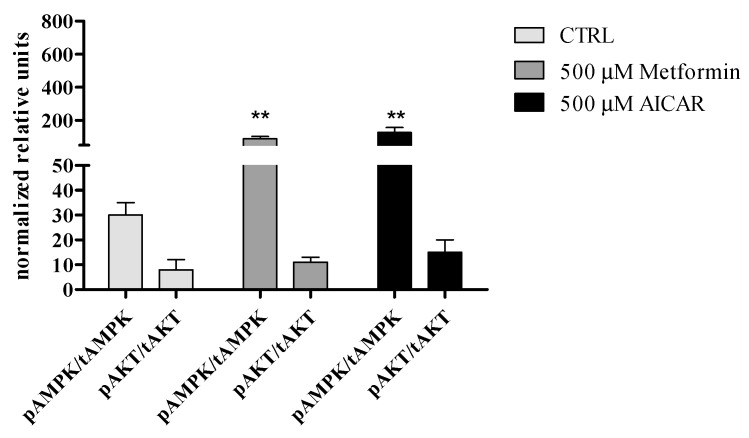
Effect of metformin treatment on AMP-activated protein kinase (AMPK α) and serine-threonine protein kinase AKT phosphorylation in rASCs. Mean and error bars (standard deviation values) were calculated based on six measurements, each performed in triplicate. Indicated differences were significant at *p* < 0.01(**).

Obtained results indicate that metformin might significantly enhance AMPK activation in undifferentiated rASC, however it does not affect the status of AKT. It seems that metformin and AICAR modulates AMPK and AKT phosporylation in rASCs in the same manner (Figure 8).

### 3.3. Osteogenic Effect of Metformin-In Vivo

#### 3.3.1. The Circulating Concentration of Osteogenic Proteins and Leptin 

**Figure 9:**
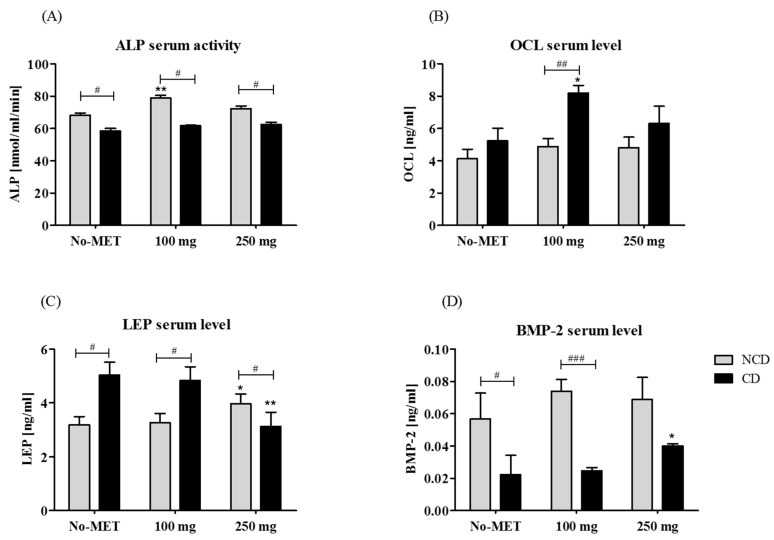
The circulating levels of ALP (**A**), OCL (**B**), LEP (**C**), and BMP-2 (**D**). Statistical analysis was performed to compare protein serum concentration in animals with (CD) and without (NCD) a cranial defect, as well as to determine the influence of metformin administration on particular serum protein levels. Differences statistically significant between NCD and CD groups were indicated with a hashtag (# *p* < 0.05; ### *p* < 0.001). The results of statistical analysis also included comparisons between animals from the control group (not receiving metformin) and metformin-treated. In these groups statistically significant differences were indicated with an asterisk (* *p* < 0.05; ** *p* < 0.01).

The activity of ALP in serum was determined using colorimetric assay, while the concentration of OCL, LEP, and BMP-2 was measured with a specific ELISA test. Obtained results revealed that ALP activity was decreased in rats that underwent craniotomy and was not altered by metformin treatment. In animals without a cranial defect the treatment with 100 mg metformin was associated with an elevated level of ALP (Figure 9A). The OCL serum level increased in animals with cranial defect, however a statistically significant difference was observed only between groups supplemented with 100 mg of metformin (Figure 9B). Concentration of leptin in rat serum was affected by 250 mg of metformin. In the group without a cranial defect supplementation with metformin caused an elevation of leptin levels, while in the group with a cranial defect the administration of 250 mg of metformin reduced protein concentration (Figure 9C). Metformin supplementation in the group without a cranial defect increased BMP-2 concentration, however the observed changes were not statistically significant. Circulating BMP-2 level was decreased in rats with a cranial defect in relation to the group without a defect. In the cranial defect group a statistically significant elevation of serum BMP-2 level was noted after supplementation with 250 mg of metformin (Figure 9D). The body weight of animals receiving metformin, both in the non-cranial as well as in the cranial defect group, was reduced (Table 1).

**Figure 10:**
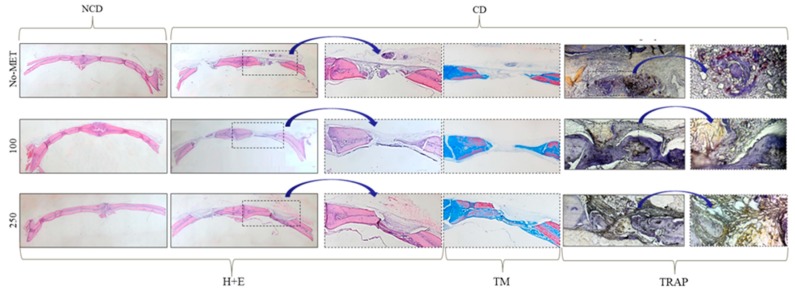
Histology of standardized cranial bone in NCD and CD group. Cranial bones of rats were harvested after four weeks of metformin treatment. Slides are from representative specimens stained with hematoxylin and eosin (H + E). Additionally, the defect sites were visualized using Masson trichrome staining (TM). The osteoclast precursors were detected using the tartrate resistant acid phosphatase (TRAP) staining.

Histological (H–E and Masson’s trichrome stain) analysis of bones revealed that metformin induces the formation of mature bone, as well as promotes new bone formation in the burr hole. Significantly enhanced bone formation was observed in animals receiving metformin at a dose of 250 mg/kg/day, both in the non-cranial defect group, as well as in the cranial defect group. In the cranial defect group, newly formed bone completely bridged defects in animals that were treated with 250 mg of metformin. The defect in animals from the control groups were occupied mainly by fibrous tissue. Moreover, the determination of the number of osteoclast precursors (TRAP-positive cells) in the reossified surface showed that metformin reduces osteoclast activity. A significant decrease was noted both in the group receiving metformin at a dose of 100 mg, as well as 250 mg/kg/day (Figure 10 and Table 2).

#### 3.3.2. Microcomputed Tomography Analysis

**Figure 11:**
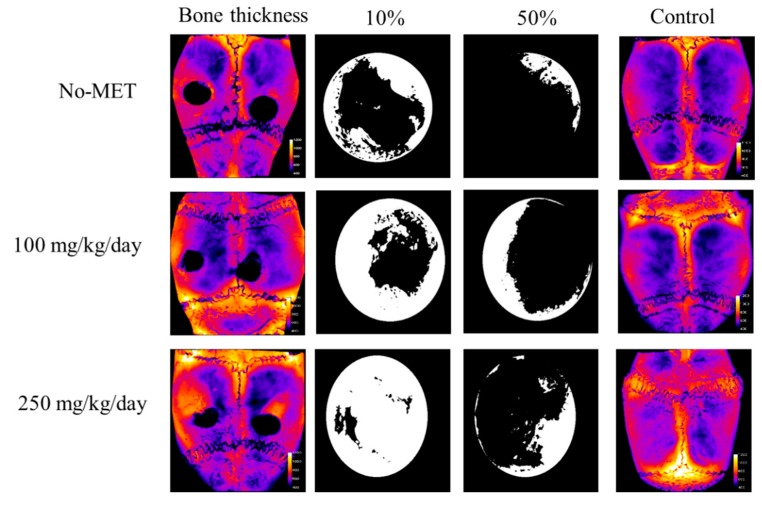
Microcomputed tomography analysis of existing and newly formed bone thickness. Bone thickness presented as heat maps (based on X-ray images)—with brighter color marking increasing bone thickness. Black and white images showing the left burr hole from the respective skull—white color shows the area of the burr hole covered by newly formed bone. 10%—burr hole covered by newly formed bone (white) which in terms of burr hole depth penetrates at least to 10% of the total burr hole depth. 50%—burr hole covered by newly formed bone (white), which in terms of burr hole depth penetrates at least to 50% of the total burr hole depth. No-met—group not receiving metformin, 100—group receiving 100 mg of metformin per day, 250—group receiving 250 mg of metformin per day.

**Figure 12:**
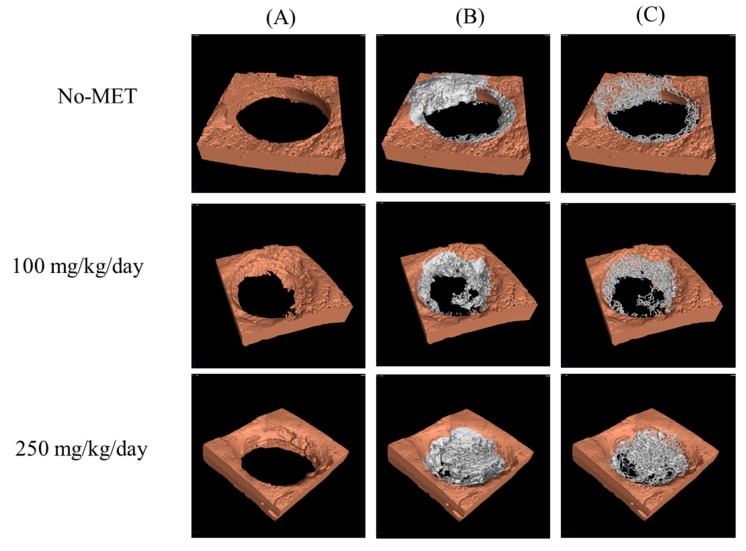
Three-dimensional uCT reconstructions of the burr holes with the new bone removed (**A**) the burr holes with the new bone overgrowing the burr hole (**B**) and trabecular skeleton of new bone showing connections between new bone trabecules (**C**). No-met—group not receiving metformin; 100—group receiving 100 mg of metformin per day, 250—group receiving 250 mg of metformin per day.

Microcomputed tomography analysis of bone thickness in the study and the control group is presented in Figure 11. Obtained images showed that in the control group bone thickness had gradually increased with the increasing dose of metformin. In the study group, the thickness of the newly formed bone in the space of bone defects was also dependent on the dose of metformin—increasing bone thickness was noted with the increase in the dose of metformin. Table 3 presents the volume of the newly grown bone in the burr hole only and the percentage coverage of the burr hole by the new bone. Animals receiving the highest dose of metformin produced the largest amount of new bone covering the burr hole. Figure 12 presents the three-dimensional µCT reconstructions of the burr holes with and without the newly formed bone, as well as the trabecular skeleton of new bone showing connections between new bone trabeculae. In all cases, the trabecular structure had the form of a network corresponding to the architecture of cancellous bone tissue. A clear pattern could be seen that with the increase of the dose of metformin also the volume of the newly formed bone increased.

## 4. Discussion

Metformin started to attract attention as an agent enhancing self-renewal and differentiation potential of progenitor cells. Pro-osteogenic effects of metformin were investigated previously, by various research groups that utilized different in vitro and in vivo experimental models [9,11,12,24]. However, these studies have shown significant discrepancies in the results and they did not resolve the underlying question whether metformin exerts a pro-osteogenic or an anti-osteogenic effect. In the current study, we have found that application of metformin in a dose of 500 µM enhances the osteogenic differentiation potential of rASCs in vitro, and what is more administration of metformin in a dose of 250 mg/kg/day promotes trabecular bone formation in a rat bilateral cranial defect model.

The majority of research concerns the influence of metformin on various osteoblast-like cell lines [18,19,41], however there are also reports focusing on the role of metformin during osteogenic differentiation of bone progenitor cells that are derived from bone marrow (BMPCs) [9,11]. In the view of bone regeneration and cell-based therapies, the aspect concerning methods of improving plasticity of progenitor cells in vitro is crucial, because the acceleration of osteogenic potential of progenitor cells may improve bone healing in vivo. Gao et al. [9] showed that metformin may promote osteogenesis in vitro of rat bone marrow derived stromal cells (BMSCs), moreover the same study revealed that metformin inhibits BMSCs adipogenic differentiation. Additionally, Molinuevo et al. [11] showed that metformin increases the ex vivo osteogenic potential of BMPCs, which was confirmed in our recent studies on a mice model [24]. In these studies, the osteogenic action of metformin was manifested by the increased expression of osteogenic markers, namely alkaline phosphatase (ALP), osteocalcin (OCL), and type I collagen (Coll-1). Moreover metformin enhanced mineralization of the extracellular matrix and promoted the formation of osteonodules in the investigated cultures [9,11].

When considering the role of metformin as an agent promoting osteogenesis of bone marrow progenitor cells in vitro, we were interested in its effects on osteoprogenitor cells within the stroma of the subcutaneous adipose tissue. The adipose-derived multipotent stromal cells (ASCs) are currently recognized as promising candidates for cell-based therapies in bone regenerative medicine, mainly due to proliferative capacity and high viability, strong secretory activity, and immunomodulatory properties, but also because of easy access to adipose tissue [42,43].

The population of rASCs used in our experiment was characterized according to the International Society of Cellular Therapy consensus [44]. We obtained adherent fibroblast-like cells, expressing specific surface markers (CD43, CD73, CD90, and CD105) and lacking the expression of CD45, therefore fulfilling all the requirements that have been suggested as the minimal criteria for MSCs defining. In the present study, we have observed that the proliferative capacity of ASCs in osteogenic cultures may be maintained by the addition of metformin [9]. At the late stage of osteogenesis, the enhanced proliferative activity was noted only in cultures treated with 500 µM of metformin. Similar observations were made for BMSCs and osteoblasts cell lines. The number of BMSCs in the osteoblastic medium increased in cultures that were treated with 100 µM metformin and the proliferative potential of the osteoblast-like cells increased in response to metformin treatment (25–500 µM) [18]. Additionally, the obtained results confirmed previous observations that the proliferation of osteoprogenitors increases during the early stage of osteogenesis, but significantly decreases during the late phase of osteogenesis [45,46]. In our experiment, the osteogenic potential of ASCs was assessed using quantitative methods regarding gene expression (qPCR) and secretome profile (ELISA) analysis. The increase of transcripts for osteogenesis markers i.e., ALP, OCL, and BMP-2 was noted in cultures treated with 500 µM and 1000 µM of metformin. These results are in good agreement with reports of Kanazawa et al. who revealed metformin—dependent increase of osteogenic factors expression in a model applying osteoblastic MC3T3-E1 cells [19]. Additionally, we showed that metformin in the concentration range of 50 µM to 1000 µM may increase the transcript level for Runt-related transcription factor 2 (Runx-2). However, recently Wang et al. showed that metformin at concentration equal 10 µM can induced osteogenic differentiation of pluripotent stem cell-derived mesenchymal stem cells (iPSC-MSCs) [47]. In this model, low concentration of metformin not only significantly stimulated alkaline phosphatase activity, but also increased the expression of Runx-2 as well as late marker of the osteoblast i.e., osterix. 

Analysis of secretory activity of rASCs revealed that 500 µM of metformin significantly stimulates ALP activity and osteocalcin production. Again, similar secretion patterns were noted in BMSCs in osteogenic cultures with metformin [11]. Paracrine effects are essential during bone regeneration, because they may facilitate the migration and differentiation of resident precursors. We determined the expression and secretion of two pro-osteogenic molecules that regulate the actions of mesenchymal stem cells i.e., BMP-2 and leptin. BMP-2 stimulates the expression of structural proteins of bone, namely collagen type I and osteocalcin, and it influences the mineralization of bone matrix [48]. Bearing in mind increased mRNA expression of BMP-2 in cultures treated with 500 µM of metformin, we expected that secretion of this protein will be also enhanced. In turn, significantly elevated concentration of BMP-2 was noted in supernatants derived from cultures treated with 10, 50, and 100 µM of metformin, as characterized by decreased mRNA level of BMP-2. It seems that there is no correlation regarding the influence of metformin on BMP-2 expression at mRNA and protein levels in rASCs. The influence of metformin on BMP-2 expression in the osteoblasts line was determined only regarding its mRNA level. Our results indicate the importance of both mRNA and protein expression studies—obviously combination of these data provide more detailed and comprehensive insight of investigated processes [49], but furthermore—in the case of MSCs it should not be neglected, as their therapeutic effects strictly depends on secretory ability. Leptin, a hormone that is produced by adipose tissue also draws attention as a modulator of osteogenesis, both in vitro and in vivo [50,51]. Our results indicate that the expression of leptin increased both at an mRNA level, as well as at the protein level in rASCs osteogenic cultures that were treated with 100 and 500 µM of metformin, while the most recent study by Zheng et al. [52] indicates the role of leptin during osteogenesis in BMSCs derived from osteoporotic rats. Several studies have shown that metformin decreases the circulating leptin level, but some others have shown evidence to the contrary [53]. For example, it was revealed that plasma leptin level is not influenced by the presence of type 2 diabetes mellitus, or short-term treatment with diet and/or sulphonylurea or metformin, but it has direct relation to glycaemic control [54]. There is also the hypothesis that metformin increases leptin sensitivity and that the anorexic and leptin-reducing effects of metformin are a result of increased leptin sensitivity [55]. Described above findings suggest that there must be a complex interaction between metformin and leptin that cannot be fully explained. In our opinion, this mechanism should be further investigated, especially in relation to the adipose-derives multipotent stromal cells. 

The osteoblastic maturation of rASCs in cultures with metformin was also confirmed by the analysis of extracellular matrix mineralization. Measurement of Alizarin Red intensity revealed that the improved deposition of calcium compounds is observed in cultures treated with 50 µM, 100 µM, and 500 µM of metformin. Nevertheless, the greatest number of nodules rich in calcium and phosphorous deposits was noted in cultures treated with metformin at a concentration of 500 µM. These cultures were also distinguished by larger nodules. Again, the obtained results are in good agreement with previous studies on rat BMSCs and osteoblasts cell lines, showing the formation of osteonodules after metformin treatment [11,18]. The majority of studies have shown that metformin acts in dose-dependent manner in terms of modulating the expression of bone-turnover markers [9,10,11,12,47]. However, in our study, we did not showed the dose-dependent effect of metformin toward rASCs. In fact, we were able only to indicate the most effective concentration of metformin that promoted the osteogenic differentiation of rASCs, i.e., 500 µM. 

Our further analysis concerned the potential involvement of AMPK and Akt in the metformin-mediated osteogenesis of rASCs. Obtained results indicate that metformin activates AMPK, however it does not alter the phosporylation status of Akt in rASCs. The involvement of AMPK signaling in osteogenesis was emphasized previously [11,56]. Despite the fact that Akt and AMPK have different cellular roles, common downstream targets are described, for example, the endothelial nitric-oxide synthase (eNOS), which, when active may be regulated by metformin [19] Moreover, Pantovic et al. [21] showed that AMPK may control the osteogenic differentiation of human dental pulp mesenchymal stem cells through late activation of the Akt/mTOR signaling axis. 

Results of our in vitro studies showing pro-osteogenic effect of metformin in rASCs cultures, were reflected in the results that were obtained using the in vivo model of rat bilateral cranial defect. We observed that metformin caused an increase in bone mass and volume, especially at a dose of 250 mg/kg per day, both in the cranial defect (CD) and the non-cranial defect group. The obtained results are in good agreement with previous studies. Using parietal bone defect (NCD) group. Molinuevo et al. [11] demonstrated that administration of metformin causes an increase in bone reossification at the lesion site, both in diabetic and nondiabetic rats. Subsequent experiments, performed by the same group of researchers additionally showed the biofunctionality of metformin osteogenic action, exhibiting its protective role in the combined therapy with rosiglitazone—an insulin-sensitizer that reduces bone mass and increases fracture risk [12]. In contrast, the studies performed by Jeyabalan et al. [25] indicated that metformin is an agent that does not promote fracture healing and has no major effect on bone mass in vivo in rodents.

We have found that metformin decreased osteoclast precursors’ activity at the lesion site. This particular aspect of our studies is not consistent with Molinuevo et al. observation [11], who noted the increased activity of TRAP-positive cells at the lesion site after metformin treatment. However, our finding supports the view that the osteogenic effect of metformin may be associated with its role as an agent that prevents osteoclasts differentiation [57]. When determining the osteogenic properties of metformin, we also evaluated its impact on serum levels of osteogenic markers. We noted that ALP serum activity in animals from the CD group was not altered by the supplementation with metformin, while concentration of OCL increased, however a significant effect was noted only in animals receiving metformin in a dose of 100 mg/kg/day. In turn, significantly elevated ALP activity was noted in animals from NCD group receiving 100 mg/kg/day, and OCL serum level was not affected by metformin supplementation. The production of ALP and OCL during the formation of endochondral and membranous bone is asynchronous. That is why the obtained results may be related to the different bone remodeling status in both animal groups—ALP is considered as an earlier differentiation marker while OCL expression is a late bone marker. Hegazy et al. [26] showed that the serum level of ALP and OCL is not influenced by metformin, therefore metformin was considered as a neutral agent that has neither an osteogenic or an anti-osteoporotic effect. Our results indicate that the analysis of bone turnover markers serum concentration is vital, however in the context of clinical relevance biochemical monitoring of bone metabolism should be correlated with other parameters of bone remodeling such analysis of bone structure, for example, using magnetic resonance images [58]. 

The serum leptin level significantly increased in response to supplementation with metformin in a dose of 250 mg/day/kg in CD group, while in the NCD group, the same dose significantly decreased the circulating level of leptin. Leptin serum level was analyzed in relation to body weight of investigated animals. Administration of metformin both in the NCD, as well as the CD group caused a significant decrease in body weight of the rats. The effects of metformin on body weight loss were observed previously. Clarifying the association of circulating leptin levels in the cranial-defect group in regards to bone healing suggests that this relationship is complex, however the results of Watanabe et al. suggested that the decrease in serum leptin but with no reduction in body fat mass, caused by another anti-diabetic drug—troglitazone—may be associated with preventing bone loss in type 2 diabetic patients [58].

Collected and discussed literature indicates the significant divergence in data relating to the osteogenic action of metformin [25,26,59]. For instance, Jeyabalan et al. used the same animals for the studies and tested a similar dose of metformin as we. The animals received metformin also for four weeks. Nevertheless, they used different model to study the influence of metformin on bone regeneration. They performed osteotomy and analyzed bone architecture in the right tibia, while fracture healing and callus volume were determined in the left femur. The protocol included application of external fixator system that was comprised from two metal blocks of titanium alloy linked to two cylindrical stainless steel bars. In turn, in our experiments we used the model of critical defect. Mainly, due to the fact that this particular model is described as one of the most reproducible and relevant to a clinical situation of interest. This model allows for the rapid determination of bone regeneration in nonload-bearing orthotopic site. The pro-osteogenic effect of metformin may be ambiguous and not always observed. Presumably, the acceleration or inhibition of the bone remodeling process by metformin, studied in rodent models, may be influenced by various factors, for example, the strain/sub-strain of animals, gender, age, dose, and duration of treatment, as well as the hormonal and the inflammatory states [25].

Importantly, there exists evidence supporting the positive effect of metformin on the bone healing process observed in vivo, which also finds reflection in clinical studies associating metformin treatment with a decrease in fracture risk in diabetic patients [17].

## 5. Conclusions

In conclusion, our results strongly weigh in favor of the pro-osteogenic effect of metformin, shedding light on its potential application in bone regenerative medicine, both as an agent that could enhance osteoblast lineage differentiation of adipose-derived multipotent stromal cells, and as a medication improving the self-renewing potential of bone tissue. 

## Figures and Tables

**Table 1:** Animal weight after 5 weeks of treatment with or without metformin in investigated groups.

Group		Body Weight of Animal (g)
**No-MET**	NCD	395 ± 5
**100**		361 ± 12 *^#^
**250**		304 ± 29 *^#^
**No-MET**	CD	364 ± 5
**100**		345 ± 3 *
**250**		345 ± 6 *

The differences between the control and experimental groups were indicated with an asterisk (*). Statistically significant differences noted between animals treated with 100 mg and 250 mg/kg/day of metformin were indicated with a hashtag (#). Observed differences were significant at *p* > 0.05. No-MET—group not receiving metformin, 100—group receiving 100 mg of metformin per day, 250—group receiving 250 mg of metformin per day. NCD—non-cranial defect group, CD—cranial defect group.

**Table 2:** Results of the histomorphometric analysis. The effect of metformin oral administration on cranial defect healing.

Characteristic	CD		
	No-MET	100 mg/kg/day	250 mg/kg/day
**Newly formed bone area/Total defect area (%)**	3 ± 1.61	28.9 ± 7.80 **	32.9 ± 9.63 **
**Newly formed bone area (mm^2^)**	0.02 ± 0.01	0.23 ± 0.06 **	0.26 ± 0.08 **
**TRAP area/Reossification area (%)**	14.59 ± 1.03	9.95 ± 2.47 **^#^	5.58 ± 2.48 **^#^

Sections of decalcified bone were stained with trichrome and quantitated using ImageJ software. Values are expressed as mean ± SD. The differences between the control and the experimental groups were indicated with a double asterisk ** and significant at 0.01. No significant differences in cranial defect healing were noted between animals treated with 100 mg and 250 mg, however the significantly reduced activity of TRAP-positive cells was noted in the group receiving 250 mg of metformin (statistically significant difference at p level equal 0.05, indicated with a hashtag).

**Table 3:** Volume of newly grown bone in the burr hole, as well as the percentage of burr hole covered by the new bone in 10 and 50% depth.

Group	V (mm^3^)	r (mm)	S10/S0 (%)	S50/S0 (%)
No-MET_L	0.59	1.13	43.3	11.0
No-MET_R	0.41	1.12	41.2	9.5
100_L	0.68	1.18	68.3	32.6
100_R	0.59	1.1	53.1	18.9
250_L	1.02	1.14	91.9	25.0
250_R	0.71	1.23	69.6	15.5

## Supplementary Materials

The following are available online at http://www.mdpi.com/2077-0383/7/12/482/s1, Figure S1: The ALP activity in non-OG and OG of rASCs cultures after metformin treatment. Values are expressed as mean ± SD, Table S1: The sequences of primers used in the experiment.
