# Supplementary material for: Metformin Promotes Osteogenic Differentiation of Adipose-Derived Stromal Cells and Exerts Pro-Osteogenic Effect Stimulating Bone Regeneration
Source: J Clin Med. 2018 Nov 26;7(12):482. doi: 10.3390/jcm7120482 (PMC6306720; doi:10.3390/jcm7120482)
Supplement: Supplementary file 1 [file jcm-07-00482-s001.pdf]

Supplementary material

Table 1. The sequences of primers used in the experiment.

Gene	Abbreviation	Primer	Sequence 5'-3'	Loci	Amplicon lenght [bp]	Accesion no.
Gyceraldehyde-3-Phosphate Dehydrogenase	GAPDH	Forward	GCACAGTCAAGGCTGAGAATG	241-262	143	NM_402691727
		Reverse	ATGGTGGTGAAGACGCCAGTA	383-363		
Alkaline phosphatase	ALP	Forward	AACGTGGCCAAGAACATCATCA	297-318	127	NM_013059.1
		Reverse	TGTCCATCTCCAGCCGTGTC	423-404		
Osteocalcin	OCL	Forward	GGTGCAGACCTAGCAGACACCA	29-50	173	NM_013414.1
		Reverse	AGGTAGCGCCGGAGTCTATTCA	201-180		
Leptin	LEP	Forward	CTGTGGCTTTGGTCCTATCT	76-95	200	NM_013076.3
		Reverse	TCCATCTTGGACAAACTCAG	275-256		
Bone Morphogenetic Protein 2	BMP-2	Forward	GAGAACACCCGGAGAAGGAGG	126-146	273	XM_006235073.1
		Reverse	AGCAGCCTCAACTCAAACTCG	398-378		
Runt-related transcription factor 2	Runx-2	Forward	CCTCAGTGATTTAGGGCGCA	963-982	153	NM_001278483.1
		Reverse	GGTGGGGAGGATTGTGTCTG	1115-1096		

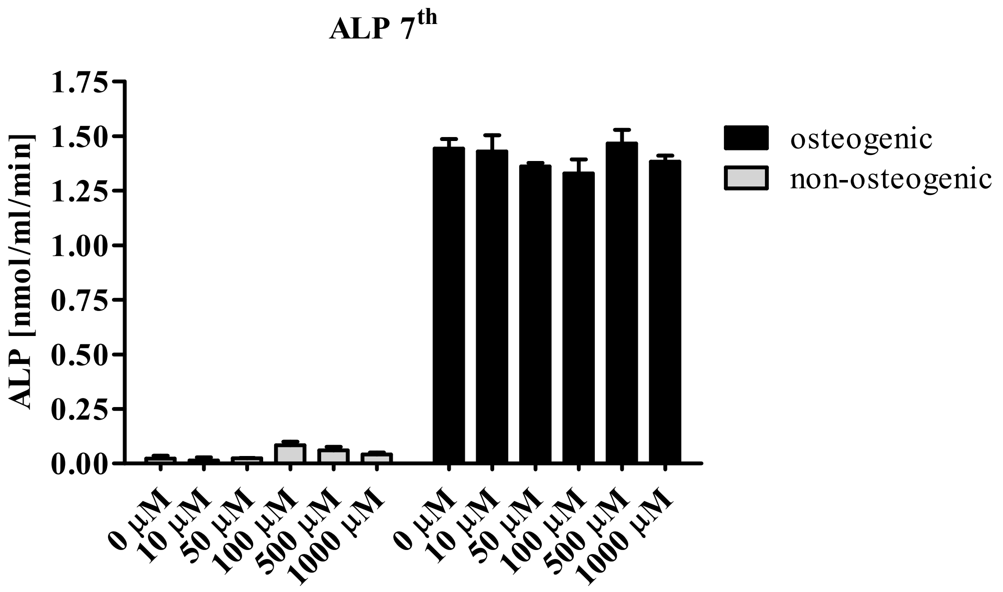

Figure 1. The ALP activity in non-OG and OG of rASCs cultures after metformin treatment. Values are expressed as mean±SD.
